# Effects of structured exercise regime on Glycosylated Hemoglobin and C reactive protein in patients with Gestational Diabetes Mellitus - A randomized controlled trial

**DOI:** 10.12669/pjms.36.7.2488

**Published:** 2020

**Authors:** Wardah Ajaz Qazi, Muhammad Naveed Babur, Arshad Nawaz Malik, Ruqia Begum

**Affiliations:** 1 Dr. Wardah Ajaz Qazi, PhD in Rehabilitation Sciences, Foundation University Institute of Rehabilitation Sciences, Foundation University Islamabad, Pakistan; 2 Prof. Dr. Muhammad Naveed Babur, PhD in Rehabilitation Sciences. Dean, Faculty of Rehabilitation Sciences, Isra University, Islamabad, Pakistan; 3 Prof. Dr. Arshad Nawaz Malik, PhD in Rehabilitation Sciences, Riphah college of Rehabilitation & Allied Health Sciences, Riphah International University, Islamabad, Pakistan; 4 Ruqia Begum, MS-OMPT. Foundation University Institute of Rehabilitation Sciences, Foundation University Islamabad, Pakistan

**Keywords:** Aerobics, C reactive protein, Exercise, Gestational diabetes mellitus, Glycosylated hemoglobin

## Abstract

**Objectives:**

To evaluate the effects of structured exercise regime on Glycosylated hemoglobin and C reactive protein in patients with gestational diabetes mellitus.

**Methods:**

This two arm parallel randomized controlled trial was conducted at Fauji Foundation Hospital, Rawalpindi from November 2018 till December 2019 on the 54 diagnosed gestational diabetes mellitus patients (Dropped out=4 Analyzed= 50) with age 20 to 40 years and gestational age from 20 to 36 weeks. Selection was done via convenient sampling technique and randomized into two groups (n=25) by sealed envelope method. Structured exercise regime group received combination of moderate intensity aerobics, stabilization and pelvic floor muscles exercises twice a week for 5 weeks (40 min per session) along with dietary and medical interventions while control group received only medical and dietary interventions with postural education. Demographics, glycosylated hemoglobin and C reactive protein were recorded at baseline then after 5 weeks of intervention. Analysis was done by SPSS 20.

**Results:**

Mean age was 35.92 ± 5.24 years in control group while 34.36 ± 5.21 years in interventional group. Between group analysis for HbA1c showed no significant difference at base line (p >0.05) but showed significant difference (p <0.05) after five weeks’ interventions. Similarly, for C reactive protein both groups showed no significant difference (p >0.05) at baseline but after five weeks of interventions showed significant difference (p<0.05).

**Conclusion:**

Structured exercise regime helps in reducing values of glycosylated hemoglobin and C reactive protein in patients with gestational diabetes mellitus.

**Trial Registration:** ClinicalTrials.gov; NCT04146740.

## INTRODUCTION

Gestational Diabetes Mellitus is a clinical as well as a public health issue now due to its highly increasing rate globally.^1^ Major contributing factors identified are obesity, family history of Type-2 Diabetes mellitus, sedentary life style and elderly mothers.^2^ Literature shows three times high risk of GDM development in patients with high serum c reactive protein levels in early pregnancy.^3^

A cohort study done in March 2019 to assess levels of C reactive protein during first trimester found its elevated levels and on follow up 9% of those women developed GDM.^4^ Another study from Pakistan reported significantly higher levels of CRP (69%) in mothers who developed GDM as compare to mothers who did not develop GDM.^5^ Similarly literature shows higher levels of HbA1c in GDM as compare to non GDM patients with high sensitivity (91.3%).^6^ A study done in Pakistan in July 2019 found obesity and deranged glycated Hemoglobin in mothers with GDM.^7^

A systematic review on effects of supervised exercise and physical activity counselling during pregnancy showed significant reduction in blood glucose levels as compare to patients who were only on usual antenatal care.^8^ A study done on obese pregnant women to assess the effects of physical activity on inflammaroty marker like hsCRP found significant difference in reducing CRP values but they didn’t mention specific dosage of exercise in physical activity which should be incorporated to treat obese gestational women having GDM.^9^ Therefore, this study was designed to assess the effects of specific combination of structured exercise with specific dosage on glycemic marker like HbA1c and inflammatory marker like C reactive protein (CRP). Furthermore the studies have given evidence that early exercises in pregnancy can be used to reduce risk of GDM development in second or third trimester^10^ but how the exercise after development of GDM during second and third trimester can effect glycemic control is the rationale of this study.

## METHODS

This parallel two arm Randomized Controlled Trial was conducted at Fauji Foundation Hospital, Rawalpindi from November 2018 till December 2019. Ethical review committee approval (Letter no. FF/FUMC/215/Phy/18) was taken from Foundation University and permission letter from Fauji Foundation Hospital prior data collection. Clinical trial registration was done with NIH USA with number NCT04146740. Written informed consent was taken from all participants.

Inclusion criteria was women with age of 20 to 40 years and gestational age between 20 to 36 weeks who have diagnosed Gestational diabetes mellitus through oral glucose tolerance test (OGTT) and were able to do 6 minutes’ walk test under severity level of 6 on the Borg scale of breathlessness. While prediabetics. patients taking insulin, patients with uncontrolled pregnancy induced hypertension, history of miscarriages and premature rupture of membrane were excluded from the study. If any included participant develop these complications later in follow up will get dropped from the study. Sample size was calculated by using open epi tool while keeping confidence interval (CI) at 95%, power at 80% with the percent of exposed with outcome is 40% and found to be 27 in both experimental (SER) and control (CTL) groups.^11^ Patients were selected through non probability convenience sampling technique and randomized into two groups via sealed envelope method.

Both groups were receiving medical and dietary interventions by obstetricians including oral hypoglycemic drugs like Tab Glucophage 500 mg tds and diet plans including less carbohydrates and more proteins. Participants in Structured Exercise Regime (SER) group was receiving exercise regime under the supervision of female physical therapist in the obstetrics ward and female gym in the rehab department along with home plan. Total 3 sessions per week for individual patient was designed for five weeks duration. It consisted of moderate intensity aerobics by using stationary cycle of dosage 3-5 METS (preset in machine) for 10 minutes and brisk walk for 10 minutes with the combination of Stabilization exercise of one set of 10 repetitions and Pelvic Floor Muscle training of one set of 20 repetitions. On the other hand, CTL i.e. active comparator group did not receive structured exercise regime they only got postural education and back care counselling from physical therapists. For primary outcome measures HbA1c and CRP, blood samples were taken by nursing staff and sent to pathology laboratory of Fauji Foundation Hospital where they were tested and verified by lab technicians and pathologists using Seimens Dines EXL 200, Beckman Coulter DxC 700 AU for HbA1c and microplate Reader Platos R-496 for CRP.

Their lab values were recorded at baseline then after treatment of five weeks on self-structured questionnaire including demographics, gravida, para and family history of patients and analyzed using SPSS version 20. Demographics were reported in the form of frequencies, percentages, mean and standard deviation. On applying Shapiro Wilk test of normality it was found that data for HbA1c was not normally distributed at baseline as probability was less than 0.05 ( p value =0.001) and was highly skewed similarly for CRP it was also not normally distributed as p value=0.001 with moderate skewness. Hence for between group analysis non parametric test i.e. Mann-Whitney U test was used.

## RESULTS

Total enrolled patients were 54 out of which two were dropped out from SER group while two from CTL group due to premature births (02) and uncontrolled Hypertension (02), leaving 25(50%) patients in both groups. Family history in SER group was positive in 13(52%) while negative in 12(28%). Although 9(36%) had positive family history and 16(64%) had negative in CTL group. In SER group 20(80%) were housewives while 5(20%) were working women similarly 18(72%) were housewives and 7(28%) were working women in CTL group.

Analysis between CTL and SER groups for HbA1c showed no significant difference (p-value=0.09) at base line but after interventions of 5 weeks’ result showed significant difference between two groups (p-value=0.002). For CRP, analysis between five weeks’ sessions of structured exercise there is significant difference between two groups (p value=0.001).

## DISCUSSION

Structured exercise regime is a non-invasive therapeutic option for preventing and managing GDM that can be readily applied to the antenatal population. This study was done to evaluate biochemical markers like glycosylated Hemoglobin (HBA1c) and C reactive protein (CRP) with antenatal exercise so that proper life style strategies should be defined for GDM patients with evidence. Many previous studies have identified the role of exercise for GDM but how the structured exercise effects inflammation and insulin resistance was recommended.^10^ The results of this study showed significant difference in the values of HbA1c and CRP between two groups after interventions applied for five weeks. These are in agreement with the literature regard to the importance of physical training during pregnancy as a non-pharmacological therapeutic tool showing significant reduction of C reactive protein p<0.05.^12^

**Table-I:** Demographics Distribution between SER Group and CTL Group.

Variables	SER Group	CTL Group
Age (years)	34.36 ± 5.21	35.92± 5.24
Gravida	4.28± 2.03	04.40± 1.89
Para	3.64 ± 1.61	2.60± 1.29

SER: Structured Exercise Regime Group, CTL: Control Group

**Table-II:** Group Analysis at Baseline and after five weeks for HbA1c & CRP

Variables	Groups		Mann Whitney U test		

		n	Mean Rank	IQR	P-value
HbA1c (Baseline)	SER	25	30.74	1.2	0.09
	CTL	25	20.26	0.9	
HbA1c (After 5 weeks)	SER	25	19.04	0.9	0.002
	CTL	25	31.96	0.9	
CRP (Baseline)	SER	25	23.88	5370	0.432
	CTL	25	27.12	1888	
CRP (After 5 weeks)	SER	25	18.68	4707	0.001
	CTL	25	32.32	960	

HbA1c: Glycosylated Hemoglobin, SER: Structured Exercise Regime Group, CTL: Control Group, CRP: c reactive protein

The study by Nobles et al in 2015 did not find statistical difference after exercise intervention done during second trimester for GDM management through individually made program of 12 weeks although this study found significant statistical difference of exercise intervention program of five weeks, performed during second and third trimester on patients having GDM.^13^ The study of Bo S et al. reported the effects of exercises and behavioral recommendations on HBA1c and CRP. The results of their study showed significant reduction in of CRP (p=0.001) and HBA1c (p<0.001) as well as triglycerides and neonatal\maternal complications in Exercise group as compared to behavioral recommendations but fasting glucose level did not show significant change.^14^ In this study we performed supervised exercise regime with specific set of intensity and duration which showed significant reduction on the level of HBA1c and CRP. A systematic review for assessing maternal and fetal outcomes after exercise in GDM mothers included 11 RCTs comparing intervention groups with groups having usual standard care. They found significant reduction in fasting blood glucose levels and post prandial glucose concentration (average standardised mean difference (SMD) -0.59, 95% CI -1.07 to -0.11; four RCTs, 363 women; I^2^ = 73%; T^2^ = 0.19) but they did not include any RCT affecting insulin resistance and inflammatory marker after exercise intervention. They recommended further researches to use different types of exercise and to evaluate its short and long term outcomes on mothers. This study focused on finding effects of planned structured aerobic exercise on maternal outcomes by reducing insulin resistance and inflammation with GDM.^15^ In the study carried out in Netherland, Oostdam N applied Fit for two exercise program in women with GDM and reported the effect on blood glucose, insulin sensitivity and birth weight. The exercise intervention performed in these patients did not show any effect on fasting blood glucose, HBA1c and birth weight most probably because of low compliance.^16^

Another RCT done to assess effects of life style interventions including physical activity and diet on metabolism and inflammation found low median levels for hsCRP in the intervention group from 28 to 30 weeks of gestation (8.3 mg/l in PA+D group, P=0.03; and 8.8 mg/l in PA group, P=0.02) as compare to control group. They used pedometer and found 11000 steps reduced hsCRP 21% in intervention group this present study also found low median level for CRP in intervention group with aerobics exercise but the number of steps and physical activity level was not calculated.^9^ Although the study by Ehrlich SF. supports the current study in which structured exercises with moderate to vigorous intensity on GDM patients during first trimester reduced the risk of GDM.^17^ A study by Johnson ST. used 24 weeks exercise program specially designed for patients with GDM improved their physical activity level from moderate to vigorous but they recommended considering all the barriers these women face. This study followed the patients for only 5 weeks although found positive results clinically^18^ but we didn’t observe all the problems and their respective solutions of GDM women in this study.

### Limitations of the study

Some patients followed the exercise plan at home only after initial sessions from hospital which could not be monitored by physical therapist secondly it was a single setting study.

## CONCLUSION

Structured exercise regime helps in reducing values of HbA1c and CRP in patients with gestational diabetes mellitus after 20 weeks gestation till 36 weeks so it can be a part of multidisciplinary management of GDM.

### Authors’ Contribution:

**WAQ:** Conception, design and conduct of study as well as data collection, and manuscript writing. He is also responsible and accountable for the accuracy or integrity of the study.

**MNB:** Did critical review, and final approval of manuscript.

**ANM:** Did data analysis, overall guidance and supervision.

**RB:** Contributed to design, data collection and data analysis.
